# Pharmacologic reversion of epigenetic silencing of the *PRKD1* promoter blocks breast tumor cell invasion and metastasis

**DOI:** 10.1186/bcr3460

**Published:** 2013-08-23

**Authors:** Sahra Borges, Heike Döppler, Edith A Perez, Cathy A Andorfer, Zhifu Sun, Panos Z Anastasiadis, E Aubrey Thompson, Xochiquetzal J Geiger, Peter Storz

**Affiliations:** 1 Department of Cancer Biology, Mayo Clinic Comprehensive Cancer Center, Mayo Clinic, Jacksonville, FL, 32224, USA; 2 Hematology/Oncology Department, Mayo Clinic, Jacksonville, FL 32224, USA; 3 Division of Biomedical Statistics and Informatics, Department of Health Sciences Research, Mayo Clinic, Rochester, MN 55905, USA; 4 Laboratory Medicine and Pathology, Mayo Clinic, Jacksonville, FL 32224, USA

**Keywords:** Decitabine, Invasion, Metastasis, PKD1, Protein kinase D1

## Abstract

**Introduction:**

DNA methylation-induced silencing of genes encoding tumor suppressors is common in many types of cancer, but little is known about how such epigenetic silencing can contribute to tumor metastasis. The *PRKD1* gene encodes protein kinase D1 (PKD1), a serine/threonine kinase that is expressed in cells of the normal mammary gland, where it maintains the epithelial phenotype by preventing epithelial-to-mesenchymal transition.

**Methods:**

The status of *PRKD1* promoter methylation was analyzed by reduced representation bisulfite deep sequencing, methylation-specific PCR (MSP-PCR) and *in situ* MSP-PCR in invasive and noninvasive breast cancer lines, as well as in humans in 34 cases of “normal” tissue, 22 cases of ductal carcinoma *in situ*, 22 cases of estrogen receptor positive, HER2-negative (ER+/HER2-) invasive lobular carcinoma, 43 cases of ER+/HER2- invasive ductal carcinoma (IDC), 93 cases of HER2+ IDC and 96 cases of triple-negative IDC. A reexpression strategy using the DNA methyltransferase inhibitor decitabine was used *in vitro* in MDA-MB-231 cells as well as *in vivo* in a tumor xenograft model and measured by RT-PCR, immunoblotting and immunohistochemistry. The effect of PKD1 reexpression on cell invasion was analyzed *in vitro* by transwell invasion assay. Tumor growth and metastasis were monitored *in vivo* using the IVIS Spectrum Pre-clinical In Vivo Imaging System.

**Results:**

Herein we show that the gene promoter of *PRKD1* is aberrantly methylated and silenced in its expression in invasive breast cancer cells and during breast tumor progression, increasing with the aggressiveness of tumors. Using an animal model, we show that reversion of *PRKD1* promoter methylation with the DNA methyltransferase inhibitor decitabine restores PKD1 expression and blocks tumor spread and metastasis to the lung in a PKD1-dependent fashion.

**Conclusions:**

Our data suggest that the status of epigenetic regulation of the *PRKD1* promoter can provide valid information on the invasiveness of breast tumors and therefore could serve as an early diagnostic marker. Moreover, targeted upregulation of PKD1 expression may be used as a therapeutic approach to reverse the invasive phenotype of breast cancer cells.

## Introduction

Breast cancer is one of the most common cancers in the United States and the leading cause of cancer-related death in women worldwide [1]. Despite vast improvement in the overall survival rate of patients with noninvasive breast cancer, advanced metastatic breast cancer remains a life-threatening disease. One of the main challenges in mammary cancer research is now to identify key proteins modulating tumor invasion, which can serve as early markers for invasive tumors as well as new drug targets.

The serine/threonine kinase protein kinase D1 (PKD1) in normal ductal epithelial cells of the breast maintains the epithelial phenotype and prevents epithelial-to-mesenchymal transition (EMT), an initial step required for cells to become motile and invasive [2]. Because local invasion is a necessary first step in metastatic dissemination to distant organs, the potential of cells to undergo EMT also defines the metastatic potential of the tumor [3-5]. In addition to its inhibitory effects on EMT, PKD1 negatively affects directed cell migration by blocking actin reorganization processes at the leading edge of migrating cells [6-11]. Furthermore, the expression and activity of PKD1 regulate the invasiveness of breast cancer cell lines by inhibiting the expression of multiple matrix metalloproteinases (MMPs) [12]. In breast cancer, PKD1 may be a key protein that inhibits the invasive phenotype, since a knockdown of PKD1 expression by reverse genetics has been shown to increase the invasiveness of the non- or minimally motile MCF-7 cells. Moreover, highly invasive MDA-MB-231 cells that do not express PKD1 were found to become noninvasive when active PKD1 was expressed [12].

Published transcriptional microarray data profiling over 350 advanced breast tumors tissues have shown a dramatic decrease of *PRKD1* gene expression in most tumor cases [13-16]. These data are in accordance with significantly reduced PKD1 expression detected in human cases of invasive ductal carcinoma (IDC) and metastatic IDC compared to samples of normal breast epithelium [12]. However, no data are available on how PKD1 expression is negatively regulated during breast tumor progression.

Aberrant epigenetic regulation of genes is one of the earliest and most frequent alteration in cancer cells and can lead to dramatic changes in cell phenotype and contribute to breast carcinogenesis [17]. Different types of genes are silenced by this manner, including tumor suppressor genes, DNA repair genes or genes that suppress invasion and metastasis [18]. In contrast to genetic mutations, epigenetic modifications such as DNA methylation are reversible and represent very promising therapeutic targets for breast cancer treatment.

The goal of this study was to determine if epigenetic silencing of *PRKD1* occurs in invasive cancer and whether this can be a driver of breast cancer cell metastasis. By comparing normal and tumor patient tissue as well as normal, noninvasive, and highly invasive breast cancer cell lines, we show that *PRKD1* gene promoter methylation directly correlates with the loss of PKD1 expression and the invasive potential of breast tumors or cells. We further show that the DNA methyltransferase inhibitor decitabine reverts *PRKD1* promoter methylation and increases PKD1 protein levels. By comparing control to PKD1-knockdown cells in an orthotopic animal model, we demonstrate that local invasion and breast cancer metastasis to the lung are specific to loss of PKD1 and can be blocked with decitabine.

## Methods

### Cell lines, antibodies and reagents

All cells lines were obtained from the American Type Culture Collection (Manassas, VA, USA). MCF-7, MDA-MB-231, MDA-MB-468 and T47D cells were maintained in Dulbecco’s modified Eagle’s medium (DMEM) with 10% fetal bovine serum (FBS). BT-20 cells were maintained in Eagle’s minimal essential medium with 10% FBS, 2 mM L-glutamine, 1.5 g/L sodium bicarbonate, 0.1 mM nonessential amino acids (NEAAs) and 1 mM sodium pyruvate. ZR-75-1 cells were maintained in RPMI medium with 10% FBS. BT-474 cells were maintained in DMEM with 10% FBS, 10 mM 2-[4-(2-hydroxyethyl)piperazin-1-yl]ethanesulfonic acid, 1% penicillin/streptomycin, 0.5 μg/ml hydrocortisone, 0.1 mM NEAAs and 10 ng/ml epidermal growth factor (EGF). MCF-10A cells were maintained in DMEM/Ham’s F-10 medium (50:50 vol/vol) with 5% horse serum, 20 ng/ml EGF, 0.5 μg/ml hydrocortisone, 100 ng/ml cholera toxin, 10 μg/ml insulin and 1% penicillin/streptomycin. NEAAs were obtained from Mediatech (Herndon, VA, USA), EGF from Pepro Tech (Rocky Hill, NJ, USA), insulin and hydrocortisone from Sigma-Aldrich (St Louis, MO, USA). Anti-β-actin antibody was obtained from Sigma-Aldrich, anti-Ki-67 from Dako (Carpinteria, CA, USA), anti-cleaved poly(ADP-ribose) polymerase (PARP) from Cell Signaling Technology (Danvers, MA, USA), anti-COX-2 from Cayman Chemical (Ann Arbor, MI, USA), anti-vimentin from EMD Millipore (Billerica, MA, USA) and anti-pS738/742-PKD from Abcam (Cambridge, MA, USA). The rabbit polyclonal antibody for PKD2 was purchased from Upstate Biotechnology (Charlottesville, VA, USA), and the mouse monoclonal antibody for PKD3 was obtained from Abnova (Walnut, CA, USA). The mouse monoclonal antibody specific for PKD1 was raised by Creative Biolabs/Creative Dynamics (Shirley, NY, USA) against a 21-amino acid peptide (KSPESFIGREKRSNSQSYIG) in the N-terminal of human PKD1, which is not present in PKD2 and PKD3. Secondary horseradish peroxidase (HRP)-linked antibodies were obtained from Roche Applied Science (Indianapolis, IN, USA). 5-aza-2′-deoxycytidine (decitabine) was purchased from EMD Millipore. Luciferin was obtained from Gold Biotechnology (St Louis, MO, USA).

### Lentiviral shRNA expression and shRNA constructs

Specific lentiviral expression constructs for short hairpin RNA (shRNA) targeting human PKD1 have been described previously [6,12] and are commercially available from Sigma-Aldrich (MISSION shRNA Plasmid DNA). Constructs used were NM_002742.x-2498s1c1 (labeled as 2) and NM_002742.x-1556s1c1 (labeled as 1). Lentivirus was produced in HEK293FT cells using the ViraPower Lentiviral Expression System (Life Technologies, Carlsbad, CA, USA). MDA-MB-231 cells were infected with PKD1-shRNA lentivirus to generate stable cell lines. After infection, cell pools were selected using puromycin (1 μg/ml) for 15 days.

### Cell lysates and Western blot analysis

Cells were washed twice with ice-cold phosphate-buffered saline (PBS) (140 mM NaCl, 2.7 mM KCl, 8 mM Na_2_HPO_4_, 1.5 mM KH_2_PO_4_, pH 7.2) and lysed with buffer A (50 mM Tris•HCl, pH 7.4, 1% Triton X-100, 150 mM NaCl, 5 mM ethylenediaminetetraacetic acid, pH 7.4) plus protease inhibitor cocktail (Sigma-Aldrich). Lysates were used for Western blot analysis as described previously [6].

### Migration and invasion assays

Transwell migration and invasion assays were performed as described previously [6,19]. Briefly, transwell chambers were coated with Matrigel (2 μg/well; BD Biosciences, San Jose, CA, USA), dried overnight and rehydrated for 1 h with 40 μl of tissue culture media. MDA-MB-231 cells were harvested, washed once with media containing 1% bovine serum albumin (BSA) and resuspended in media containing 0.1% BSA, then 10,000 cells were seeded onto the transwell insert (10^5^ cells). NIH-3T3-conditioned medium served as a chemoattractant in the lower chamber. Remaining cells were used to analyze the expression of genes of interest. After 16 hours, cells on top of the transwell insert were removed and cells that had migrated to the lower surface of the filters were fixed in 4% paraformaldehyde, stained with 4′,6-diamidino-2-phenylindole and counted. For impedance-based real-time chemotactic assays, cells were seeded onto a CIM-Plate 16 transwell from Roche Applied Science. After attachment, cell migration or invasion (coating of top well with 2 μg of Matrigel) toward NIH-3T3-conditioned media was continuously monitored in real time for the indicated times using the xCELLigence RTCA DP Instrument (Roche Applied Science).

### RT-PCR

Cellular RNA was isolated using RNA-Bee (Tel-Test, Friendswood, TX, USA) according to the manufacturer’s instructions and transcribed into cDNA using the ImProm-II Reverse Transcription System (Promega, Madison, WI, USA). For the transcription reaction, 1 μg of oligo d(T)_18_ primer (New England Biolabs, Beverly, MA, USA) and 1 μg of RNA were incubated in a total volume of 10 μl at 70°C for 10 min. Next, 5× buffer, 40 U of RNAsin Plus RNase Inhibitor (Promega), 200 μM deoxyribonucleotide triphosphate (New England Biolabs) and 1 μl of ImProm-II reverse transcriptase (Promega) were added to a total volume of 20 μl. Samples were then incubated for 5 min at 25°C, and the reaction was carried out at 42°C for 60 min and then heat-inactivated at 70°C for 15 min. The resulting cDNA pool was subjected to polymerase chain reaction (PCR) analysis using specific primer sets. Primers used for human PKD1 were 5′-TTCTCCCACCTCAGGTCATC-3′ and 5′-TGCCAGAGCACATAACGAAG-3′. The primers used for glyceraldehyde 3-phosphate dehydrogenase (GAPDH) were 5′-TCAACGGATTTGGTCGTATTG-3′ and 5′-AGAGTTAAAAGCAGCCCTGGTGA-3′. PCR reactions were carried out under the following conditions: 1 min at 55°C and a 1-min extension at 72°C for 35 cycles.

### Quantification of *PRKD1* gene and exon expression levels

PKD1 mRNA expression was measured as described previously [20]. Briefly, double-stranded cDNA were synthesized using the total RNA from each cell line. PCR primers were designed using the template regions recommended by SnowShoes-FTD. The gene expression levels were calculated as the sum of the individual exon read counts and exon junction read counts. The expression levels of genes and exons were normalized using the total aligned reads from the sample and the length of the exon or gene (reads per kilobases per million).

### Patient samples, tissue microarrays and immunohistochemistry

Biospecimens were obtained and processed from the Mayo Clinic Tissues Registry under protocols 09–001642, 09–001599, 09–000530 and 11–001638 and approved by the Mayo Clinic Institutional Review Board (IRB) and the Institutional Biosafety Committee. The IRB approved a waiver of specific informed consent in accordance with 45 CFR § 46.116 as justified by the investigator. As a limited data set was used and a data use agreement had been completed, in accordance with 45 CFR § 164.514, HIPAA authorization (Health Insurance Portability and Accountability Act of 1996, Pub L 104–191, 110 Stat 1936) was not required. Tissue microarray (TMA) sections were deparaffinized (1 h at 60°C), dewaxed in xylene (five times for 4 min each time) and gradually rehydrated with ethanol (100%, 95% and 75%, twice with each concentration for 3 min). The rehydrated TMA sections were rinsed in water and subjected to hematoxylin and eosin staining or to antigen retrieval in citrate buffer (pH 6.0) as described by the manufacturer (Dako). Slides were treated with 3% hydrogen peroxide (5 min) to reduce endogenous peroxidase activity and washed with PBS containing 0.5% Tween 20. Proteins of interest were detected using specific antibodies diluted in PBS-Tween 20 and visualized using the EnVision+ Dual Link Labelled Polymer Kit following the manufacturer’s instructions (Dako). Images were captured using the Aperio ScanScope scanner (Aperio, Vista, CA, USA).

### Reduced representation bisulfite deep sequencing

Analysis of CpG island methylation by reduced representation bisulfite deep sequencing was determined as described previously [21]. Briefly, DNA (2 mg) extracted from cell lines was fragmented using endonuclease MspI, followed by QIAquick purification (QIAGEN, Valencia, CA, USA). Digested DNA was then treated according to the Illumina protocol (Illumina, San Diego, CA, USA), separated by 2% agarose gel and purified using the QIAquick Gel Extraction Kit (QIAGEN). The purified DNA was modified and purified using the EpiTect Bisulfite Kit (QIAGEN). The bisulfite-converted DNA was then amplified by PCR. The amplification conditions were as follows: 5 min at 95°C, 30 s at 98°C, then 66 cycles (10 s at 98°C, 30 s at 65°C, 30 s at 72°C), followed by 5 min at 72°C. The PCR product was purified using the MinElute PCR Purification Kit (QIAGEN), and the concentration of a final library was measured using the Agilent 2100 Bioanalyzer (Agilent Technologies, Santa Clara, CA, USA). The library was sequenced on an Illumina Genome Analyzer IIx sequencing instrument according to standard Illumina cluster generation and sequencing protocols. Methylated C base was measured by counting the C/C+T ratio. Summarized methylation data on the *PRKD1* promoter CpG island were obtained by averaging all CpG sites. These data represent the percentage of methylated CpGs over total number of CpGs in the island. The differentially methylated CpG islands were identified using the limma software package as described for analysis of gene expression. A *P*-value cutoff of 0.05 was applied for significantly methylated CpG islands.

### Bisulfite conversion and methylation-specific PCR

Genomic DNA was isolated from cell lines and tumor samples using the QIAamp DNA Mini Kit (QIAGEN) according to the manufacturer’s instructions. Genomic DNA (1 μg) was then modified with a sodium bisulfite solution using the EZ DNA Methylation Kit (Zymo Research, Irvine, CA, USA) and amplified by PCR using the GC Rich PCR Amplification-Advantage GC 2 Polymerase Mix and PCR Kit (Clontech Laboratories, Mountain View, CA, USA). The primers specific for the methylated *PRKD1* gene promoter were 5′-AGAGGGTTAGTCGGGTAGC-3′ and 5′-ACGTCCGCGAAATAACTTA-3′, and for the unmethylated *PRKD1* gene promoter, they were 5′-TTTAGGTTGATTTGTAGATGGAAT-3′ and 5′-CAATCCACTACTACCCATAACAA-3′. Conditions for amplification were as follows: 1 min at 94°C, 35 cycles (30 s at 94°C, 45 s at 50°C and 1 min at 72°C), followed by a final extension at 72°C for 10 min. PCR products were analyzed on a 1.5% agarose gel and visualized by ethidium bromide staining.

### *In situ* methylation-specific PCR

*In situ* methylation-specific PCR (MSP-PCR) was performed as described previously [22]. Paraffin-embedded sections were digested with pepsin (2 mg/ml in 0.1 M HCl) (Dako) for 20 min, washed in water for 1 min and air-dried. Sections were then placed in 3 M bisulfite solution, heated at 94°C for 3 min and incubated at 50°C for 15 h. The *in situ* MSP-PCR step was performed as follows: denaturation at 94°C for 1 min, amplification for 35 cycles (94°C for 1 min and 50°C for 1.5 min) using AmpliTaq Gold 360 DNA Polymerase Kit (Applied Biosystems, Carlsbad, CA, USA). The primers used for specific *in situ* amplification of the methylated *PRKD1* gene promoter were: 5′-GGATTTTGAGGTTCGGAAC-3′ and 5′-CAAATTCTTAACGACGACGA-3′. After amplification, *in situ* hybridization was performed using the internally biotin-labeled probe (1 μg/ml) specific for the methylated *PRKD1* promoter, 5′-AATTCTTAACGACGACGACG-3′, diluted with *in situ* hybridization buffer (Enzo Life Sciences, Farmingdale, NY, USA). The PCR product and the probe were codenatured at 95°C for 8 min and hybridized at 37°C for 15 h. Sections were then washed in 0.2× saline sodium citrate solution (0.15 M NaCl, 0.015 M sodium citrate, pH 7) with 2% BSA for 5 min, incubated with HRP-conjugated streptavidin for 2 hours (1:50; R&D Systems, Minneapolis, MN, USA) and exposed to 3,3′-diaminobenzidine (DAB) (Dako) at room temperature. Samples were then counterstained with eosin to stain negative cells pink in contrast to the brown DAB signal. Images were captured using the Aperio ScanScope scanner and analyzed using the Aperio Positive Pixel Count algorithm in ImageScope software (Aperio).

### Orthotopic tumor model and treatment

Animal experiments were performed under protocol A17313 approved by the Mayo Clinic Institutional Animal Care and Use Committee. Female nonobese diabetic severe combined immunodeficiency (NOD *scid*) mice were anesthetized, and MDA-MB-231 cell lines additionally expressing luciferase were injected into the fourth mammary gland on the right side of each animal. A total of 500,000 cells washed three times in PBS and mixed with 30 μl of complete Matrigel (BD Biosciences) were injected. Mice were treated with 5 mg/kg decitabine diluted in a saline solution (Sigma-Aldrich) or saline solution alone according to the timeline shown in Figure 4A. Decitabine or control saline solution was delivered by intraperitoneal injection. Body weight and tumor volume (caliper measurement) were determined once per week. The presence of metastases was detected using the IVIS Spectrum Imaging System (PerkinElmer, Waltham, MA, USA). At the end point, primary tumors and sites of metastases were removed and analyzed as indicated.

### Statistical analysis

GraphPad Prism version 4.0c software (GraphPad Software, Inc, La Jolla, CA, USA) was used for all statistical analyses. Statistical significance was determined using a two-tailed Student’s *t*-test and standard deviations. For all analyses, *P* < 0.05 was considered significant.

## Results

### DNA methylation of the *PRKD1* promoter silences PKD1 expression in invasive breast cancer cell lines

**Figure 1:**
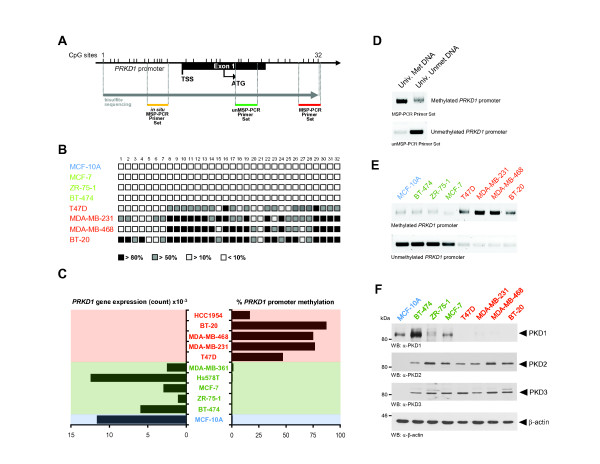
**DNA methylation of the*PRKD1*promoter silences PKD1 expression in invasive breast cancer cell lines. (A)** Schematic representation of *PRKD1* promoter region. CpG sites found by bisulfite sequencing and regions amplified by methylation-specific PCR (MSP-PCR) or *in situ* MSP-PCR are indicated. **(B)***PRKD1* promoter methylation was determined for the non- or minimally invasive breast cancer cell lines MCF-7, ZR-75-1 and BT-474; the highly invasive cell lines MDA-MB-231, MDA-MB-468, T47D and BT-20; and the normal MCF-10A cell line. Unmethylated CpG sites are shown as empty squares and methylated CpG sites as filled squares according to their percentage of methylation for all clones analyzed. **(C)** Percentage of methylation of *PRKD1* promoter was determined by bisulfite sequencing. In addition, RNA was isolated from cells, and *PRKD1* expression levels were calculated as the sum of the individual exon read counts. **(D)** Controls for MSP-PCR are shown. Enzymatically methylated or unmethylated DNA was modified by bisulfite treatment, and MSP-PCR was performed using the indicated primers. **(E)** Genomic DNA from indicated cell lines was modified by bisulfite treatment, and MSP-PCR was performed using methylation-specific primers for the *PRKD1* promoter. **(F)** Indicated cell lines were analyzed for protein kinase D1 (PKD1), PKD2 or PKD3 expression by Western blotting. Immunostaining for β-actin served as a loading control. All experiments were independently performed at least three times.

PKD1 is a kinase that negatively affects directed cell migration and invasion of tumor cells [6,9,23] and maintains the epithelial phenotype of breast cancer cells through negative regulation of EMT [2]. In human samples of IDC, PKD1 is downregulated at its protein level, but the mechanisms underlying how this is achieved are unknown [12]. So far, the only mutation in the *PRKD1* gene found for breast cancer does not explain the loss of its expression [24], and it is conceivable that downregulation of PKD1 expression is due to epigenetic modifications such as DNA methylation of its promoter [25]. The *PRKD1* gene promoter contains a large CpG island covering 1.2 kb, including the transcription start site and the entire exon 1 (Figure 1A). We assessed the methylation status over a stretch of 32 CpG sites of the *PRKD1* promoter by bisulfite sequencing in a subset of highly invasive and non- or minimally invasive breast cancer cell lines as well as in the “normal” MCF-10A cells (Figure 1B). Interestingly, very low levels of methylation (0.14% to 1.1%) were found in the “normal” MCF-10A cells and non- and minimally invasive BT-474, ZR-75-1, MCF-7, Hs578T and MDA-MB-361 cell lines, whereas most CpG sites were found to be hypermethylated (16.5% to 86.9%) in the invasive breast cancer cell lines T47D, MDA-MB-231, MDA-MB-468, BT-20 and HCC1954. Hypermethylation of the *PRKD1* promoter in breast cancer cell lines directly correlated with a loss of *PRKD1* gene expression (Figure 1C). The migratory and invasive abilities of adherent growing cells, including MCF-10A or breast cancer cell lines, were verified with impedance-based real-time migration and invasion assays using the xCELLigence System (Roche Applied Science) (Additional file 1: Figure S1). The invasive propensities of all analyzed cell lines are summarized in Additional file 2: Table S1.

Next, to confirm bisulfite sequencing analyses, we designed sets of primers that allow distinguishing between methylated and unmethylated *PRKD1* promoter (shown in Figure 1A). Both primer sets were tested using universal methylated or unmethylated DNA (Figure 1D). Using these primers sets in MSP-PCR, we confirmed that DNA methylation of the *PRKD1* promoter was present only in the highly invasive breast cancer cell lines, whereas it was unmethylated in the non- or minimally invasive cells (Figure 1E). *PRKD1* promoter methylation directly correlated with loss of PKD1 expression in highly invasive cells (Figure 1F). The two other PKD isoforms, PKD2 and PKD3, were upregulated in all breast cancer cells independently of their invasive potential, similarly to previously described findings [26]. Taken together, using different methods, we show that the methylation status of the gene promoter directly correlates not only with the loss of PKD1 expression but also with the invasive potential of breast cancer cells.

### Epigenetic silencing of the *PRKD1* gene promoter correlates with breast tumor invasiveness

**Figure 2:**
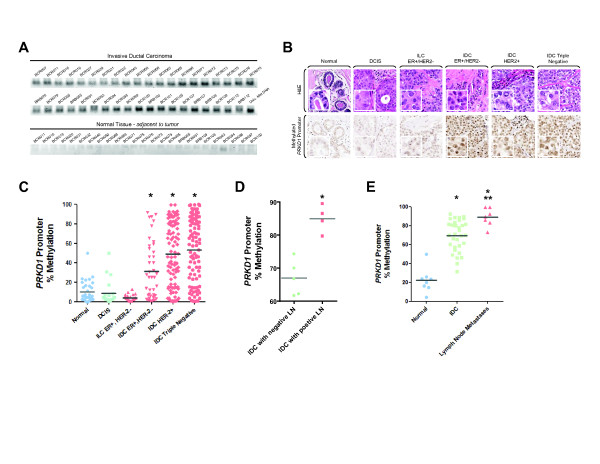
**Epigenetic silencing of the*PRKD1*gene promoter correlates with breast tumor invasiveness. (A)** Patient samples (invasive ductal carcinoma (IDC) and normal tissue) were analyzed for *PRKD1* gene promoter methylation. Genomic DNA was extracted from 25 mg of tissue and modified by bisulfite treatment. Methylation-specific PCR (MSP-PCR) was performed using methylation-specific primers for *PRKD1* promoter. Universal methylated DNA (Univ. Met. DNA) served as a positive control. All samples were prepared at the same time and were analyzed on the same agarose gel. **(B)***PRKD1* gene promoter methylation was determined in confirmed human breast cancer and normal human breast tissue by tissue microarray. DNA was bisulfite-modified *in situ*. *In situ* MSP-PCR and hybridization were performed using methylation-specific primers and probes. **(C)** Statistical analysis of *PRKD1* promoter methylation was conducted using the Aperio Positive Pixel Count algorithm in ImageScope viewing software. *P* values were acquired using Student’s *t*-test using GraphPad Prism version 5 software. *P ≤ 0.005, indicating extreme statistical significance. DCIS = ductal carcinoma *in situ*, IDC = invasive ductal carcinoma, ILC = invasive lobular carcinoma. **(D)***PRKD1* gene promoter methylation was determined in human tissue from IDC with positive or negative lymph nodes. **(E)***PRKD1* gene promoter methylation was determined in normal human breast tissue (adjacent to tumor), IDC and lymph nodes metastases. For (D) and (E), statistical analysis was performed using the Aperio Positive Pixel Count algorithm in the ImageScope viewing software. *P* values were calculated using Student’s *t*-test in GraphPad Prism version 5 software. *P ≤ 0.0001, indicating extreme statistical significance compared to “normal.” **P ≤ 0.005, indicating statistical significance compared to IDC.

We utilized our MSP-PCR method to analyze genomic DNA (gDNA) from fresh frozen tissues from patients with IDC and normal breast tissue adjacent to tumor for *PRKD1* promoter methylation. All tumor samples analyzed (*n* = 39) showed *PRKD1* promoter methylation, whereas all normal controls (*n* = 25) except one did not show any methylation (Figure 2A; same numbers for tumor and normal tissue indicates that they are from the same patient).

Since extraction of gDNA from fresh frozen tumor tissue sections can also contain gDNA from tumor-associated tissue (including stromal, fat and immune cells), we established an *in situ* MSP-PCR allowing the detection of methylated *PRKD1* promoter in formalin-fixed, paraffin-embedded tissue. The conditions were tested using MDA-MB-231 and MCF-7 cells as a positive and a negative control, respectively (Additional file 3: Figure S2). We utilized this method to specifically determine *PRKD1* promoter methylation in breast tumor cells. We analyzed the methylation status of the *PRKD1* promoter in 34 cases of “normal” tissue (from mammoplasty or adjacent to tumor), 22 cases of ductal carcinoma *in situ* (DCIS), 22 cases of estrogen receptor-positive, HER2-negative (ER+/HER2-) invasive lobular carcinoma (ILC), 43 cases of ER+/HER2- IDC, 93 cases of HER2+ IDC and 96 cases of triple-negative IDC (Figure 2B,C). Relatively low levels of promoter methylation were observed in normal (average of 9.9%) and DCIS (average of 8.5%). Samples of ER+/HER2- ILC showed a slight decrease in promoter methylation with an average of methylated cells at 4.2%. In contrast, the percentage of tumor cells positive for methylated *PRKD1* promoter significantly increased in samples from patients with ER+/HER2- IDC (average of 26.9%) and even more in HER2+ (average of 55.4%) or triple-negative (average of 59.7%) samples. Methylation of the *PRKD1* promoter correlated with loss of PKD1 protein expression in the same tissue (Additional file 4: Figure S3).

To determine if loss of PKD1 can be linked to metastasis, first we analyzed the methylation status of the *PRKD1* promoter in primary tumors from patients with IDC who were diagnosed with positive or negative dissemination to lymph nodes (Figure 2D). As predicted for invasive cancer, we detected a high percentage of positive tumor cells for methylated *PRKD1* promoter in both samples. However, the percentage of promoter methylation was significantly higher in IDC with positive lymph nodes (average of 84.95%) as compared to IDC with negative lymph nodes (average of 66.99%). Next, we compared *PRKD1* promoter methylation in normal tissue adjacent to tumor, primary tumor and lymph node metastases from patients with IDC (Figure 2E). In these samples, we observed a significant increase in the percentage of positive cells for *PRKD1* promoter methylation in primary tumors (average 71.1%) and a further increase in lymph node metastasis (average 89.1%) compared to adjacent “normal” tissue (average 22.2%). Hypermethylation of the *PRKD1* promoter correlated with loss of PKD1 expression in the same tissue (Additional file 5: Figure S4).

In summary, our analysis of patient data indicates that decrease or loss of PKD1 expression in human breast cancer is due to hypermethylation of the *PRKD1* promoter. Such silencing correlates with the invasive potential of tumors. This suggests that both PKD1 expression and methylation of its promoter could serve to determine the invasive potential of breast tumors.

### Pharmacologic inhibition of *PRKD1* methylation leads to PKD1-dependent reversion of the invasive phenotype

**Figure 3:**
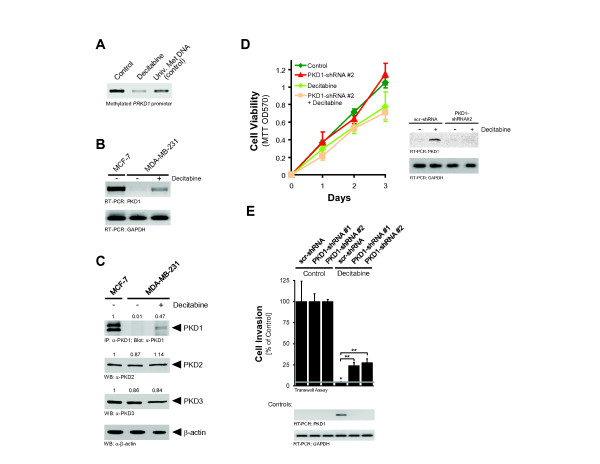
**Pharmacologic inhibition of*PRKD1*methylation leads to PKD1-dependent reversion of the invasive phenotype.** In all experiments, cells were treated with 10 μM decitabine or dimethyl sulfoxide (DMSO) as control for 3 days. **(A)** DNA from MDA-MB-231 treated cells was modified by bisulfite treatment, and methylation-specific PCR was performed with specific primers for the methylated *PRKD1* promoter. **(B)** RNA was isolated from treated cells, and RT-PCR using specific primers for protein kinase D1 (PKD1) and glyceraldehyde 3-phosphate dehydrogenase (GAPDH) mRNA expression was performed. **(C)** Lysates from treated cells were analyzed by Western blotting for expression of PKD1, PKD2, PKD3 or β-actin as a loading control. Values represent densitometry calculations (values obtained for MCF-7 cells were set to 1). Intensities of bands were calculated using Quantity One 1-D Analysis Software (Bio-Rad Laboratories, Hercules, CA, USA) and normalized to bands obtained with β-actin. **(D)** MDA-MB-231 control and PKD1 short hairpin RNA (shRNA) cells (shRNA sequence 2) were treated with 10 μM decitabine or DMSO as a control. Cell viability was determined using a tetrazolium dye assay. The results presented are the means ± SD. **(E)** MDA-MB-231 control and PKD1 shRNA cells (shown are two different shRNA sequences, 1 and 2) were treated with 10 μM decitabine or DMSO as a control and seeded onto Matrigel-coated transwell filters. Transwell invasion assays were performed over a period of 16 h. scr-shRNA, scrambled control short hairpin RNA. The results presented are means ± SD. *P* values were calculated using a two-tailed Student’s *t*-test and standard deviations. For all analyses, *P* < 0.05 was considered significant. All experiments were independently performed at least three times.

On the basis of the preceding experiments, we hypothesized that inhibition of methylation of the *PRKD1* promoter with DNA methyltransferase inhibitors can lead to reexpression of PKD1 and reversion of the invasive phenotype. To test this hypothesis, we treated MDA-MB-231 cells with decitabine (5-aza-2′-deoxycytidine) and tested its effect on *PRKD1* promoter methylation using MSP-PCR (Figure 3A). Decitabine induced the demethylation of the *PRKD1* promoter, and this correlated with the reexpression of PKD1 at the transcriptional level (Figure 3B) and at the protein level without affecting the levels of expression of PKD2 and PKD3 (Figure 3C). Similar results were obtained with two additional invasive breast cancer cell lines (Additional file 6: Figure S5).

Inhibition of methyltransferases can lead to induction of multiple genes in cancer, including the estrogen receptor [27]. To distinguish between decitabine-induced PKD1-dependent and PKD1-independent effects, we next compared control MDA-MB-231 cells (scrambled shRNA control) to cells previously infected with shRNA targeting PKD1 (PKD1-shRNA 1 or PKD1-shRNA 2). Expression of shRNA specific for PKD1 in these cells blocks decitabine-induced reexpression of PKD1 as compared to parental or control cells. Treatment with decitabine slightly decreased MDA-MB-231 cell viability, and this effect was independent of the PKD1 expression status (Figure 3D). However, the inhibitory effects of decitabine on tumor cell invasion were partially restored in PKD1-knockdown cells (Figure 3E). This suggests that the inhibitory effects of decitabine on cell invasion are due in part to *PRKD1* promoter demethylation and reexpression of PKD1. Since PKD1 was previously characterized as a negative regulator of cell motility, our data suggest that a PKD1 reexpression strategy may be used as a therapeutic approach to reduce or prevent breast cancer cell metastasis.

### PKD1-dependent and PKD1-independent effects of decitabine treatment on primary tumor size and metastatic progression

**Figure 4:**
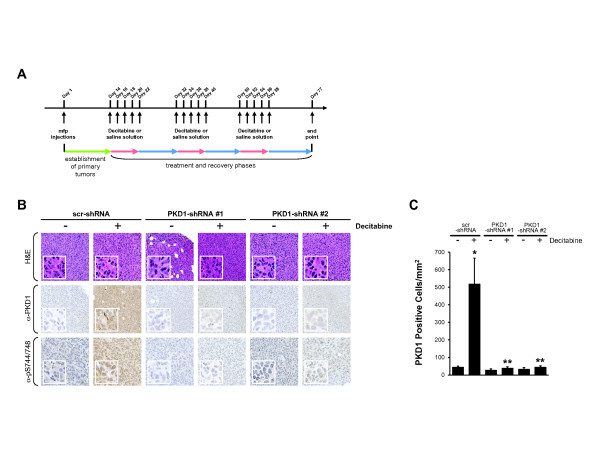
**Decitabine-induced PKD1 reexpression in primary tumors. (A)** Treatment schedule and timeline. MDA-MB-231 control and protein kinase D1 short hairpin RNA (PKD1 shRNA) cells (shRNA sequences 1 and 2) expressing luciferase were injected into the mammary fat pads of female nonobese diabetic severe combined immunodeficiency (NOD *scid*) mice. After establishment of primary tumors (days 1 to 14), mice were treated with 5 mg/kg decitabine or a saline solution injected intraperitoneally. Treatments were performed every other day over a period of 10 days. This was followed by a 10-day recovery phase. Overall, three cycles of treatment occurred, as shown in the timeline. **(B)** Analysis of PKD1 expression and PKD activity in primary tumors from mice with orthotopically implanted MDA-MB-231 scrambled control shRNA (scr-shRNA) cells and two different PKD1 shRNA lines (PKD1 shRNA 1 and 2) after control or decitabine treatment. Formalin-fixed, paraffin-embedded tissue was either stained with hematoxylin and eosin or analyzed for PKD1 expression (anti-PKD1) and PKD activity (anti-pS738/pS742) antibodies. Representative pictures of primary tumors are shown. **(C)** The number of PKD1-positive cells was calculated using the Aperio Positive Pixel Count algorithm in ImageScope viewing software. *P* values were calculated using Student’s *t*-test in GraphPad Prism version 5 software. *P ≤ 0.005, indicating statistical significance compared to saline-treated scr-shRNA cells. **P ≤ 0.005, indicating statistical significance compared to decitabine-treated scr-shRNA cells.

To test whether a decitabine-induced reexpression strategy for PKD1 can be an efficient way to treat breast tumor growth and metastasis *in vivo*, we orthotopically implanted MDA-MB-231 cells either stably expressing scrambled shRNA control or two different specific shRNA sequences for PKD1 into the mammary fat pads of female NOD *scid* mice. The efficacy of PKD1-targeted shRNA to block decitabine-induced PKD1 reexpression was verified prior the injection (not shown). After establishment of primary tumors, mice were treated with decitabine every other day (starting at day 14 after injection). Within the total of 76 days, three treatment phases with five treatments each were followed by a recovery phase (see schematic in Figure 4A). At the end points of the experiments, tumors and tissues of potential sites of metastasis were extracted. Primary tumors were analyzed by immunohistochemistry for PKD1 expression using a monoclonal antibody. As expected, decitabine-induced PKD1 reexpression was significantly blocked in tumors of mice when PKD1 shRNA cell lines were implanted (Figure 4B, C). Of note, some heterogeneity in the intensity of PKD1 expression in different areas of each tumor sample was detected, probably due to decitabine delivery to the tumor (not shown).

A significant PKD1-independent decrease of primary tumor size was noted when mice were treated with decitabine (Additional file 7: Figure S6A). This was due to a decitabine-induced decrease in cell proliferation (staining for Ki-67) and a slight increase in apoptotic cells (staining for cleaved PARP) (Additional file 7: Figure S6B). These effects were independent of the presence or absence of PKD1 and were not surprising, as suggested by our *in vitro* studies (Figure 3D).

**Figure 5:**
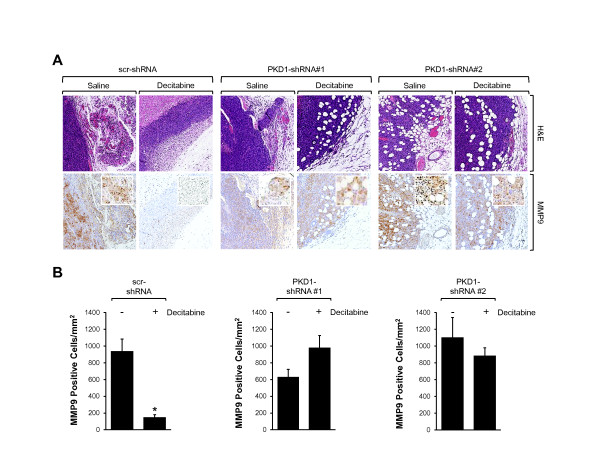
**PKD1-dependent effects of decitabine treatment on local invasion. (A)** After control (saline) or decitabine treatment, primary tumors obtained with the MDA-MB-231 scrambled control short hairpin RNA (scr-shRNA) or protein kinase D1 (PKD1) shRNA 1 or 2 cell line and were either stained with hematoxylin and eosin (H&E) or analyzed for matrix metalloproteinase 9 (MMP9) expression (anti-MMP9). Representative pictures are shown. **(B)** The number of MMP9-positive cells per square millimeter was determined using the Aperio Positive Pixel Count algorithm in ImageScope viewing software. *P* values were calculated using Student’s *t*-test in GraphPad Prism version 5 software. *P ≤ 0.005 indicates statistical significance.

When we analyzed tumor edges and connections to the mouse mammary tissue in control cells, we observed a reduced local invasion in the tumors treated with decitabine and reexpressing PKD1. However, cells expressing shRNA targeting PKD1, thus not allowing decitabine-induced reexpression, showed local invasion similar to that of untreated cells (Figure 5A). Because MMPs, and particularly MMP9, are highly expressed in epithelial cancers and are correlated with tumor cell migration and invasion of surrounding tissue [28-30], we examined MMP9 expression in orthotopic tumors. We found that MMP9 expression was significantly reduced only in the decitabine-treated control tumors (scrambled control shRNA, or scr-shRNA), but not in tumors generated with PKD1 shRNA cells or in saline-treated tumors, in which local tumor cell invasion was observed (Figure 5A, B). This suggested that observed inhibitory effects of decitabine treatment on local tumor cell invasion and primary tumor expansion are dependent on upregulation of PKD1 expression.

**Figure 6:**
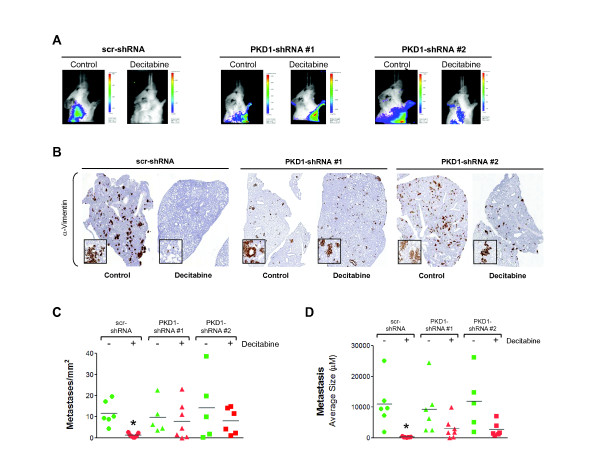
**PKD1-dependent effects of decitabine treatment on metastasis progression. (A)** The presence of metastasis was detected using the IVIS Spectrum Imaging System *in vivo* at day 77. Representative pictures from each treatment group are shown. PKD1, protein kinase D1; scr-shRNA, scrambled control short hairpin RNA. **(B)** The presence of pulmonary metastasis was detected by immunostaining with an antibody specific for human vimentin (detects human cancer cells). **(C)** The number of pulmonary metastases per square millimeter was quantified from five fields in each lung. **(D)** The area of metastatic nodules in each section was quantified from five fields in each lung. For (C) and (D), the values are means ± SEM. *P* values were calculated using a two-tailed Student’s *t*-test and standard deviation. *P ≤ 0.005 indicates statistical significance.

We next analyzed if this also affects metastasis to distant organs. Previously, COX-2 expression was associated with metastasis of breast cancer cells to lungs [31], bones [32] and brain [33]. When comparing primary tumors for COX-2 expression, we observed a significant decrease in cells expressing COX-2 in the decitabine-treated control group, but not the decitabine-treated PKD1 shRNA group (Additional file 7: Figure S6B). The MDA-MB-231 orthotopic animal model favors tumor cell metastasis to the lung [34]. Therefore, we next examined whether PKD1 reexpression induced by decitabine was able to inhibit breast tumor cell infiltration of the lungs (Figure 6A). Moreover, mice implanted with control cells and treated with saline solution had large numbers of lung metastases, whereas control mice treated with decitabine showed very few or no metastases to their lungs as determined by immunohistochemical staining for human vimentin as a marker for human cancer cells (Figure 6B, C). Analysis of the few metastases in the lungs of the scr-shRNA control mice treated with decitabine showed that they remained homogeneously small (average of 248.5 μm^2^) as compared to saline-treated mice, in which metastases to the lungs were approximately 40 times larger (average 10,926.6 μm^2^) (Figure 6D). Importantly, the block of PKD1 reexpression (PKD1 shRNA cells) significantly blocked inhibitory effects of decitabine on numbers of metastases per square millimeter and their average size (Figure 6B, C, D). Moreover, despite some heterogeneity, mice with tumors formed by cells containing PKD1 shRNA showed no statistical differences in the number of lung metastases or in their size, regardless of the treatment. This suggests that decitabine-mediated inhibition of breast tumor cell metastasis is dependent on reexpression of PKD1.

## Discussion

Depending on the cell type and the activation mechanism, PKD enzymes are involved in many biological processes including cell adhesion, vesicle transport, cell survival and cell migration (reviewed in [35]). In prostate and breast tissue, PKD1 contributes to maintenance of the epithelial phenotype by inhibiting EMT and upregulating E-cadherin expression [2,23,36]. In addition, active PKD1 negatively impacts cell migration and invasion through inhibition of actin reorganization processes at the leading edge [6,7,9-11,37,38], as well as downregulation of expression of MMPs [12].

Because of its negative regulation of cell motility, it is not surprising that downregulation of PKD1 has been described for advanced gastric [39], prostate [23,37] and breast cancers [12]. Moreover, loss of PKD1 has been associated with increased invasiveness and risk of metastases in gastric cancer and osteosarcoma [39,40]. We previously have shown the importance of PKD1 for breast cancer cell invasion by demonstrating that a knockdown of PKD1 in the low invasive breast cancer cell line MCF-7 led to an increase of its invasive potential, and reexpression of a constitutively active PKD1 in highly invasive MDA-MB-231 cells impaired their invasive phenotype [12]. We now show that breast cancer cell lines can be divided into cells that express PKD1 and cells that do not express PKD1 (Figure 1). Of note, the other two PKD isoforms, PKD2 and PKD3, were upregulated in all breast cancer cell lines independently of their invasive potential (Figure 1F). This confirms previously described data showing that these two isoforms may have tumor-promoting functions. For example, both have been shown to contribute to cell proliferation and growth of triple-negative breast cancer cells [26]. It may be speculated that to become aggressive, breast cancer cells undergo an isoform switch in PKD proteins.

The mechanisms by which PKD1 expression is silenced are not well understood. Recent studies have identified missense mutations in the coding sequence of the *PRKD1* gene in human colorectal and breast cancers [24,25]. However, these mutations do not explain the loss of PKD1 expression during the invasive progression of breast cancer, suggesting another type of regulation. Epigenetic alterations such as promoter-specific DNA methylation promote dramatic changes in gene expression and have been shown to play a critical role during tumorigenesis [17,41]. Herein we demonstrate that the silencing of PKD1 observed in invasive breast cancer cell lines, as well as in IDC, is also linked to hypermethylation of its promoter (Figures 1 and 2). Our PCR-based assay established to detect *PRKD1* gene promoter methylation in formalin-fixed tissue also allowed us to determine the methylation status of *PRKD1* specifically in ductal epithelial cells of normal breast and in tumor cells (Figure 2). Interestingly, the percentage of positive cells for *PRKD1* promoter methylation was found to be significantly increased in the most aggressive types of breast cancer, including triple-negative cancer, and, in IDC cases, gradually increased lymph nodes positive for tumor cells as well as lymph node metastases. Changes in the epigenetic regulation of gene expression, in contrast to genetic alterations, are believed to occur in a gradual rather than an abrupt manner [42]. In accordance with this, the analysis of our progression TMAs indicates that *PRKD1* promoter methylation is acquired during progression to IDC and increases when IDC become lymph node-positive. This implies that loss of PKD1 expression during breast cancer progression may contribute to mammary neoplasia and lead to the acquisition of metastatic characteristics.

Our studies also show that the silencing of *PRKD1* caused by the hypermethylation of its promoter occurs in IDC, but not in ILC (Figure 2C). This is in accord with previous studies that showed that there are clearly differences in the methylation patterns that characterize ILC and IDC, which may be the cause of the different morphology or the clinical features of these two tumor types. For example, hypermethylation of the death-associated protein kinase (*DAPK*) gene promoter was found to be significantly higher in ILC than in IDC [43], whereas the promoter of the *Twist* gene was less frequently methylated in ILC than in IDC [44]. However, both types of breast carcinoma are aggressive and invasive. At this point, we cannot explain why the *PRKD1* promoter is not epigenetically regulated by methylation in ILC. However, it is possible that PKD1 in this subtype of breast cancer may be regulated in its kinase activity.

Combining the knowledge gained from cell culture studies and data obtained with patient specimens (Figures 1 and 2), a strategy to prevent an invasive phenotype and metastasis of breast cancer cells may be reactivation of PKD1. To start testing this hypothesis, we determined whether the *PRKD1* gene can be reexpressed in invasive breast cancer and if this could reverse the invasive phenotype *in vitro* as well as *in vivo* (Figures 3, 4, 5, and 6). Reversing epigenetic silencing of genes can be achieved by applying DNA methyltransferase inhibitors such as the US Food and Drug Administration (FDA)-approved drug decitabine. However, owing to the multiple genes targeted, it is difficult to assess the specificity of such drugs. For example, treatment with decitabine induces the reexpression of multiple genes, including tumor suppressors such as *TP53* and *CDKN1A* or the gene encoding the ER [27,45]. Therefore, to assess the specific effects of decitabine-induced PKD1 reexpression on an invasive phenotype of breast tumor cells, we used our lentiviral system comprising a scr-shRNA and two different PKD1-specific shRNA sequences to prevent PKD1 reexpression [6].

In invasive breast cancer cell lines, treatment with decitabine reversed the epigenetic silencing of the *PRKD1* gene (Figure 3A through 3C and Additional file 6: Figure S5). This led to a significant decrease in MDA-MB-231 cell invasion, which was due to reexpression of PKD1 (Figure 3E). In an orthotopic model of breast cancer, treatment with decitabine showed PKD1-independent effects on primary tumor growth, probably due in part to a decrease of cell proliferation and an increase of apoptosis [45-48], as indicated by staining of Ki-67 and cleaved PARP (Additional file 7: Figure S6). However, decitabine’s inhibitory effects on local tumor invasion and metastasis to the lung were dependent on reexpression of PKD1 in this model (Figures 5 and 6). Cells reexpressing PKD1 formed not only less but also much smaller tumor colonies in the lungs (Figure 6C, D). Therefore, it is likely that PKD1 not only affects the ability of cancer cells to escape from the primary tumor and invades through the surrounding matrix and enter the bloodstream but also may impact their ability to adapt to their new environment.

Our data also support a clinical application of DNA methyltransferase inhibitors such as decitabine to prevent cancer cell invasion and metastasis. However, the clinical application of DNA methyltransferase inhibitors also raises several concerns, especially regarding their effect on the nonspecific activation of genes in normal cells as well as their potential mutagenicity. Some studies have analyzed the differential effect of such agents in normal cells as compared to tumor cells. Interestingly, normal cells were less sensitive to drug-induced gene activation, suggesting that DNA methylation is more easily reversed in the targeted tumor cells, in which abnormally methylated CpG islands are responsible for the silencing of tumor suppressor genes [49-51]. In addition, clinical trials involving decitabine have shown some promising results with negligible side effects for patients with leukemia or myelodysplatic syndrome. More important, although follow-up studies have described a slight increase of global genomic demethylation, they also have shown an absence of the development of secondary malignancy [52].

## Conclusion

Taken together, our data suggest a role for *PRKD1* promoter silencing by methylation as a measure of how the invasive potential of breast tumors is achieved or increased. They also suggest that reexpression of *PRKD1*, for example, by using DNA methyltransferase inhibitors such as the FDA-approved drug decitabine, could be an effective strategy to prevent tumor metastasis.

## Abbreviations

DCIS: Ductal carcinoma *in situ*; EGF: Epidermal growth factor; EMT: Epithelial-to-mesenchymal transition; ER: Estrogen receptor; HER2: Human epidermal growth factor receptor 2; IDC: Invasive ductal carcinoma; ILC: Invasive lobular carcinoma; PKD: Protein kinase D; TMA: Tissue microarray; TN: Triple-negative.

## Competing interests

The authors declare that they have no conflicts of interest.

## Authors’ contributions

SB and PS conceived and designed the experiments. SB, HD and ZS performed the experiments. SB, HD, ZS, PS, XJG, EAT, CAA, PZA and EAP analyzed the data. CAA, PZA, EAP and XJG contributed reagents/materials/analysis tools. SB and PS wrote the paper. All authors read and approved the final version of the manuscript.

## Supplementary Material

Additional file 1: Figure S1 Invasion and migration abilities of invasive and non-invasive breast cancer cell lines. Cell migration and invasion was measured for the indicated cell lines using the xCELLigence RTCA DP Instrument. Cells were seeded onto a CIM-Plate 16 transwell directly on transwell filters for cell migration or onto Matrigel-coated transwell filters for cell invasion measurement. After 2 h of attachment, cell migration toward NIH-3T3 conditioned medium was monitored continuously in real time over a period of 24 h. Error bars represent four experiments. Click here for file

Additional file 2: Table S1 Correlation between PRKD1 promoter methylation status, PKD1 expression and breast cancer cell line characteristics. EGFR, epidermal growth factor receptor; ER, estrogen receptor; PKD1, protein kinase D1; PR, progesterone receptor. Click here for file

Additional file 3: Figure S2 *In situ* detection of DNA methylation of the PRKD1 promoter in MCF-7 and MDA-MB-231 cells. PRKD1 gene promoter methylation was determined in MCF-7 and MDA-MB-231 cells. DNA was bisulfite-modified *in situ*. *In situ* methylation-specific PCR and hybridization were performed using methylation-specific primers and probes. Bars represent 100 μm. Click here for file

Additional file 4: Figure S3 PKD1 expression and activity in human breast cancer and normal human breast tissue. Tissue microarray slides containing histologically confirmed human breast cancer and normal human breast tissue samples were analyzed for protein kinase D1 (PKD1) expression using an isoform-specific antibody. Representative pictures of normal, ductal carcinoma *in situ* (DCIS), invasive lobular carcinoma (ILC), invasive ductal carcinoma (IDC) and triple-negative breast tumor tissue are depicted. ER, estrogen receptor; H&E, hematoxylin and eosin. Click here for file

Additional file 5: Figure S4 PKD1 expression in human invasive ductal carcinoma and metastasis from lymph nodes. Tissue microarray slides containing histologically confirmed matching human invasive ductal carcinoma (IDC), lymph node metastasis and normal human breast tissue samples were analyzed for protein kinase D1 (PKD1) expression using an isoform-specific antibody. Representative pictures of normal, IDC and lymph node metastasis tissues are depicted. Click here for file

Additional file 6: Figure S5 Decitabine-induced reexpression in T47D and HCC1954 breast cancer cell lines. Cells were treated with decitabine (10 μM) or control as indicated for 3 days. RNA was isolated and RT-PCR using specific primers for protein kinase D1 (PKD1) and glyceraldehyde 3-phosphate dehydrogenase (GAPDH) expression was performed. Click here for file

Additional file 7: Figure S6 PKD1-independent effects of decitabine treatment on primary tumor growth. (A) Volume of primary mammary fat pad tumors obtained with MDA-MB-231 cells stably expressing scrambled control shRNA (scr-shRNA), protein kinase D1 (PKD1) shRNA 1 and PKD1-shRNA 2 after control treatment (saline) or treatment with decitabine (as indicated in Figure 4A). Volume was determined by caliper measurement and is shown as percentage of control. *P ≤ 0.005. (B) Immunohistochemical analysis of above primary orthotopic mammary fat pad tumors for the expression of Ki-67, cleaved poly(ADP-ribose) polymerase (PARP) and COX-2. Representative pictures of primary tumors are shown. Click here for file
