# Supplementary material for: Pharmacologic reversion of epigenetic silencing of the PRKD1 promoter blocks breast tumor cell invasion and metastasis
Source: Breast Cancer Res. 2013 Aug 23;15(2):R66. doi: 10.1186/bcr3460 (PMC4052945; doi:10.1186/bcr3460)
Supplement: Additional file 1: Figure S1 — Invasion and migration abilities of invasive and non-invasive breast cancer cell lines. Cell migration and invasion was measured for the indicated cell lines using the xCELLigence RTCA DP Instrument. Cells were seeded onto a CIM-Plate 16 transwell directly on transwell filters for cell migration or onto Matrigel-coated transwell filters for cell invasion measurement. After 2 h of attachment, cell migration toward NIH-3T3 conditioned medium was monitored continuously in real time over a period of 24 h. Error bars represent four experiments. [file bcr3460-S1.pdf]

Table S1

Cell line	*PRKD1* methylation	PKD1 expression	Tumorigeni city	*in vitro* Invasiveness	Metastasis *in vivo*	ER	PR	EGFR	HER2
**MCF-10A**	N	++	N	N	N	-	-	+	+
**BT-474**	N	+++	Y + E2 pellet	P, low	N	-	+	+	+
**ZR-75-1**	N	+	Y + E2 pellet	P, low	N	+	+	+	+
**MCF-7**	N	++	Y + E2 pellet	N	N	+	-	+	-
**T47D**	P	N	Y + E2 pellet	P	N	+	+	+	-
**MDA-MB-231**	P	N	Y	P, high	Y	-	-	+	-
**MDA-MB-468**	P	N	Y	P	Y	-	-	+	-
**BT-20**	P	N	Y	P	N	-	-	+	-

N = Negative, P = Positive (expressed), Y = Yes, + = low, ++ = medium, +++ = high, - = absent

Table S1: Correlation between *PRKD1* promoter methylation status, PKD1 expression and breast cancer cell line characteristics.
