# Supplementary material for: Pharmacologic reversion of epigenetic silencing of the PRKD1 promoter blocks breast tumor cell invasion and metastasis
Source: Breast Cancer Res. 2013 Aug 23;15(2):R66. doi: 10.1186/bcr3460 (PMC4052945; doi:10.1186/bcr3460)
Supplement: Additional file 2: Table S1 — Correlation between PRKD1 promoter methylation status, PKD1 expression and breast cancer cell line characteristics. EGFR, epidermal growth factor receptor; ER, estrogen receptor; PKD1, protein kinase D1; PR, progesterone receptor. [file bcr3460-S2.pdf]

Figure S2

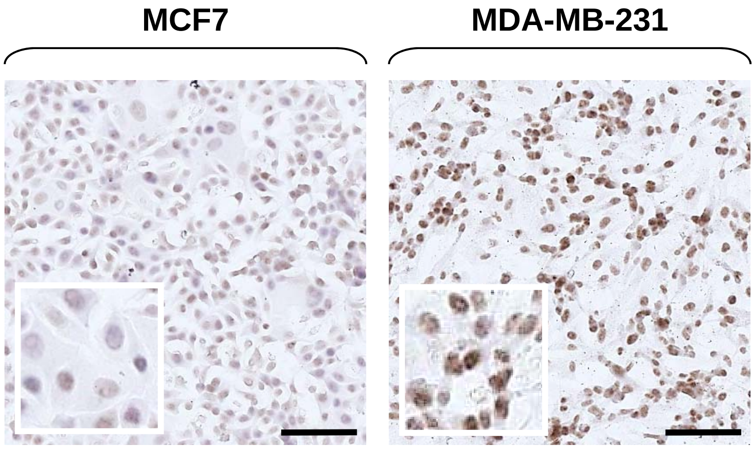

Figure S2: *In situ* detection of DNA methylation of the PRKD1 promoter in MCF7 and MDA-MB-231 cells. *PRKD1* gene promoter methylation was determined in MCF7 and MDA-MB-231 cells. DNA was bisulfite modified *in situ*. *In situ* MSP-PCR and hybridization were performed using methylation-specific primers and probes. Bars represent 100 µm**.**
