# Supplementary material for: Pharmacologic reversion of epigenetic silencing of the PRKD1 promoter blocks breast tumor cell invasion and metastasis
Source: Breast Cancer Res. 2013 Aug 23;15(2):R66. doi: 10.1186/bcr3460 (PMC4052945; doi:10.1186/bcr3460)
Supplement: Additional file 3: Figure S2 — In situ detection of DNA methylation of the PRKD1 promoter in MCF-7 and MDA-MB-231 cells. PRKD1 gene promoter methylation was determined in MCF-7 and MDA-MB-231 cells. DNA was bisulfite-modified in situ. In situ methylation-specific PCR and hybridization were performed using methylation-specific primers and probes. Bars represent 100 μm. [file bcr3460-S3.pdf]

Figure S3

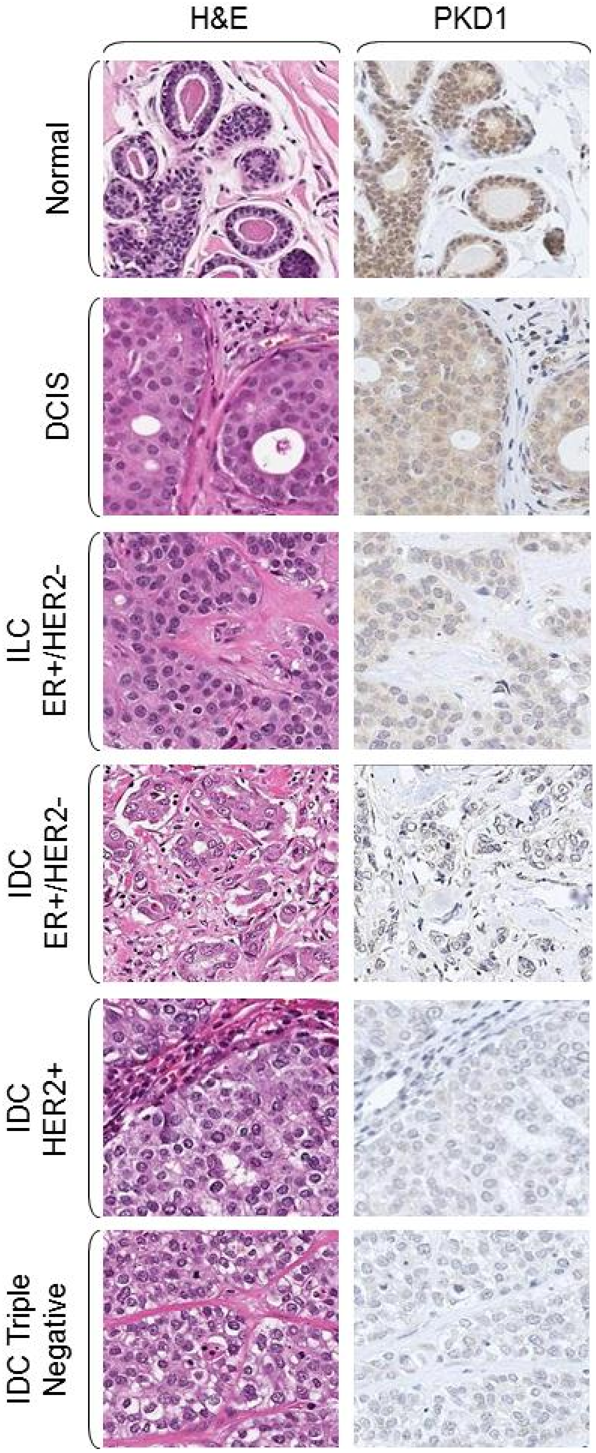

Figure S3: PKD1 expression and activity in human breast cancer and normal human breast tissue. Tissue microarray slides containing histologically-confirmed human breast cancer and normal breast tissue samples were analyzed for PKD1 expression using an isoform-specific antibody. Representative pictures of normal, DCIS, ILC, IDC and Triple Negative breast tumor tissue are depicted.
