# Supplementary material for: Pharmacologic reversion of epigenetic silencing of the PRKD1 promoter blocks breast tumor cell invasion and metastasis
Source: Breast Cancer Res. 2013 Aug 23;15(2):R66. doi: 10.1186/bcr3460 (PMC4052945; doi:10.1186/bcr3460)
Supplement: Additional file 4: Figure S3 — PKD1 expression and activity in human breast cancer and normal human breast tissue. Tissue microarray slides containing histologically confirmed human breast cancer and normal human breast tissue samples were analyzed for protein kinase D1 (PKD1) expression using an isoform-specific antibody. Representative pictures of normal, ductal carcinoma in situ (DCIS), invasive lobular carcinoma (ILC), invasive ductal carcinoma (IDC) and triple-negative breast tumor tissue are depicted. ER, estrogen receptor; H&E, hematoxylin and eosin. [file bcr3460-S4.pdf]

Figure S4

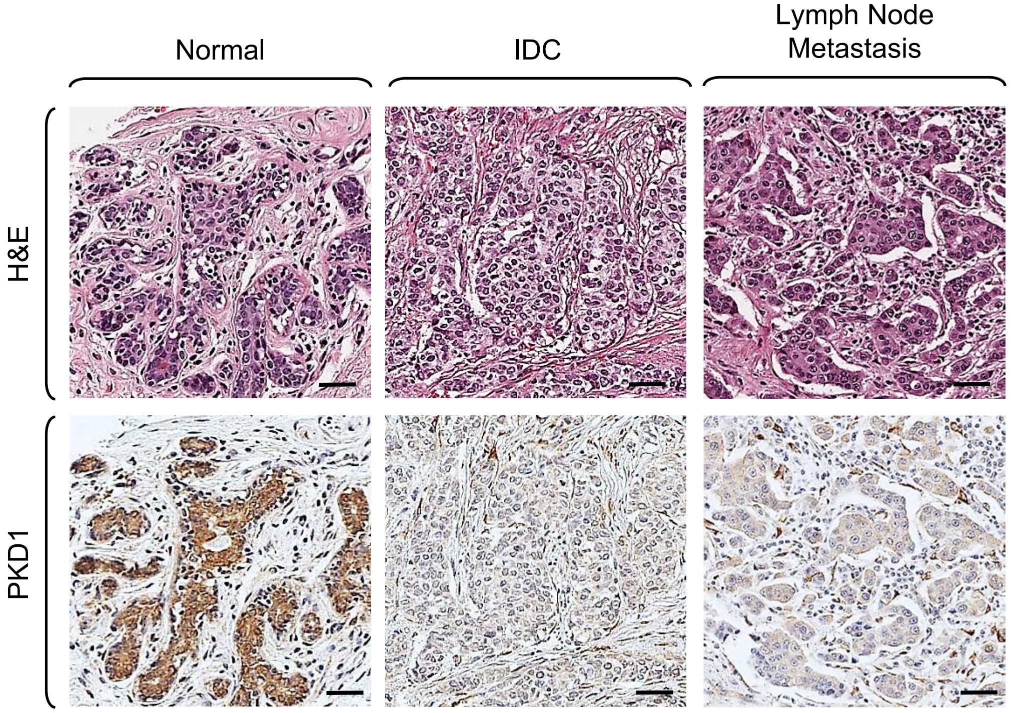

Figure S4: PKD1 expression in human IDC and metastasis from lymph nodes. Tissue microarray slides containing histologically-confirmed matching human invasive ductal carcinoma (IDC), lymph node metastasis and normal human breast tissue samples were analyzed for PKD1 expression using an isoform-specific antibody. Representative pictures of normal, IDC and lymph node metastasis tissues are depicted.
