# Supplementary material for: Pharmacologic reversion of epigenetic silencing of the PRKD1 promoter blocks breast tumor cell invasion and metastasis
Source: Breast Cancer Res. 2013 Aug 23;15(2):R66. doi: 10.1186/bcr3460 (PMC4052945; doi:10.1186/bcr3460)
Supplement: Additional file 5: Figure S4 — PKD1 expression in human invasive ductal carcinoma and metastasis from lymph nodes. Tissue microarray slides containing histologically confirmed matching human invasive ductal carcinoma (IDC), lymph node metastasis and normal human breast tissue samples were analyzed for protein kinase D1 (PKD1) expression using an isoform-specific antibody. Representative pictures of normal, IDC and lymph node metastasis tissues are depicted. [file bcr3460-S5.pdf]

*Figure S5*

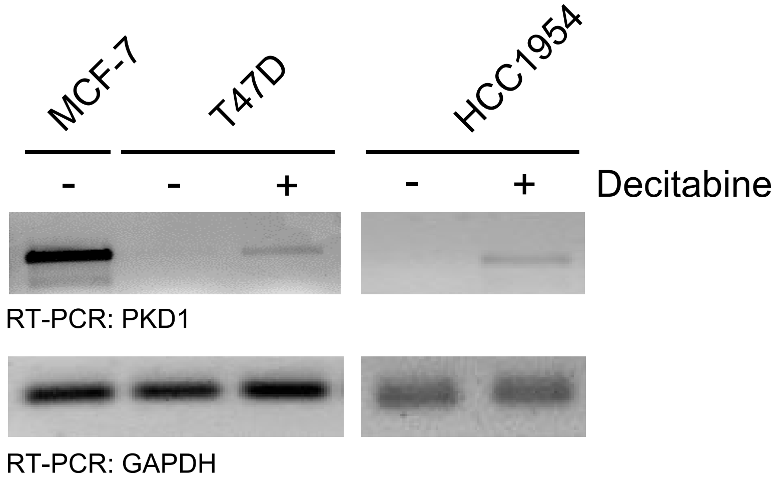


**Figure S5: Decitabine induced re-expression in T47D and HCC1954 breast cancer cell lines.**Cells were treated with decitabine (10 μM) or control as indicated for 3 days. RNA was isolated and RT-PCR using specific primers for PKD1 and GAPDH was performed.
