# Supplementary material for: Pharmacologic reversion of epigenetic silencing of the PRKD1 promoter blocks breast tumor cell invasion and metastasis
Source: Breast Cancer Res. 2013 Aug 23;15(2):R66. doi: 10.1186/bcr3460 (PMC4052945; doi:10.1186/bcr3460)
Supplement: Additional file 6: Figure S5 — Decitabine-induced reexpression in T47D and HCC1954 breast cancer cell lines. Cells were treated with decitabine (10 μM) or control as indicated for 3 days. RNA was isolated and RT-PCR using specific primers for protein kinase D1 (PKD1) and glyceraldehyde 3-phosphate dehydrogenase (GAPDH) expression was performed. [file bcr3460-S6.pdf]

Figure S6

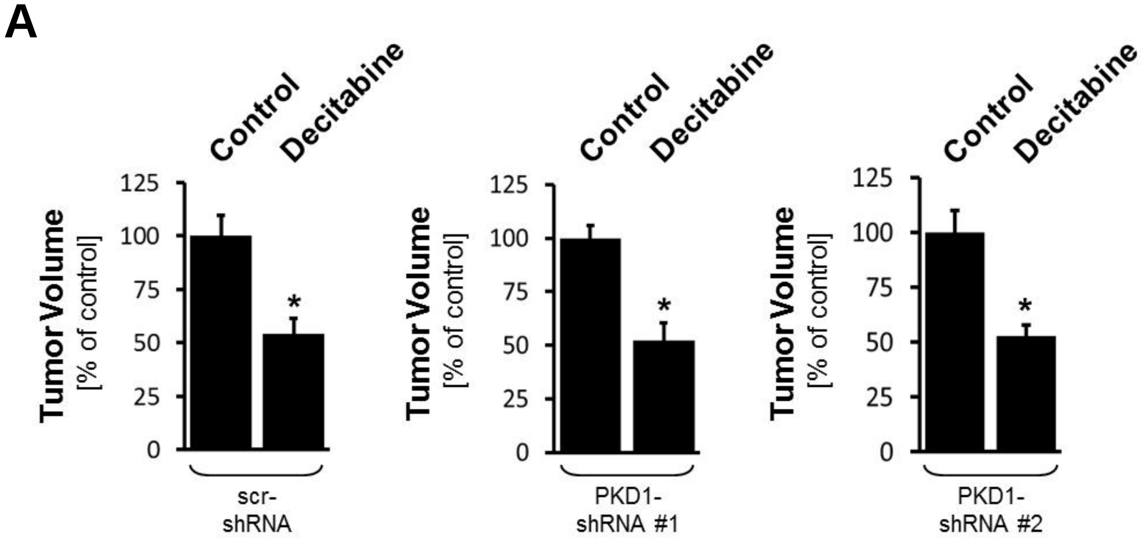

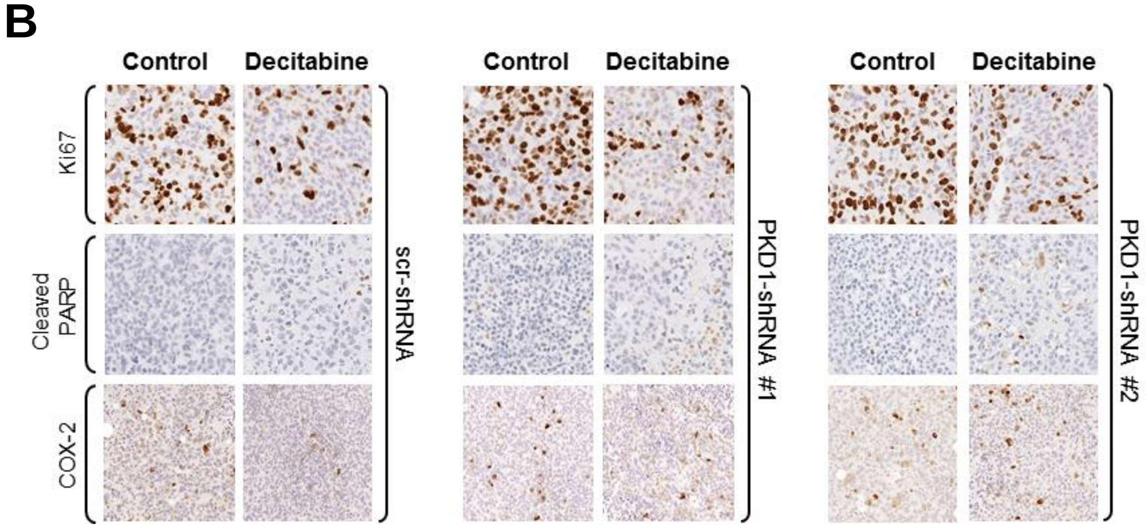

Figure S6: PKD1-independent effects of decitabine treatment on primary tumor growth. A: Volume of primary orthotopic mfp tumors obtained with MDA-MB-231 cells stably expressing control shRNA (scr-shRNA), PKD1-shRNA #1 or PKD1-shRNA #2 after control treatment (saline) or treatment with Decitabine (as indicated in Fig. 4A). Volume was determined with caliper measurement and is shown as % of control. * indicates p ≤ 0.005. **B**: IHC analysis of above primary orthotopic mfp tumors for the expression of Ki67, cleaved PARP and COX-2. Representative pictures of primary tumor*s* are depicted.
